# Detection and Characterization of Clinical *Bordetella trematum* Isolates from Chronic Wounds

**DOI:** 10.3390/pathogens10080966

**Published:** 2021-07-30

**Authors:** Christian Buechler, Claudio Neidhöfer, Thorsten Hornung, Marcel Neuenhoff, Marijo Parčina

**Affiliations:** 1 Institute of Medical Microbiology, Immunology and Parasitology, University Hospital Bonn, University of Bonn, 53127 Bonn, Germany; claudio.neidhoefer@ukbonn.de (C.N.); marcel.neuenhoff@ukbonn.de (M.N.); parcina@uni-bonn.de (M.P.); 2 Clinic and Polyclinic for Dermatology and Allergology, University Hospital Bonn, University of Bonn, 53127 Bonn, Germany; thorsten.hornung@ukbonn.de

**Keywords:** *Burkholderiales*, *Alcaligenaceae*, *Bordetella trematum*, non-fermenting Gram-negative bacilli, wound infection, chronic wounds, diabetic ulcer, antibiotic resistance

## Abstract

*Bordetella trematum* is a relatively newly discovered and potentially frequently overlooked *Bordetella* species, mostly isolated from chronic wounds and predominantly in those of the lower extremities. Its susceptibility profile and clinical significance is still debated, given the limited amount of available data. We contribute providing a molecular and phenotypical analysis of three unique clinical *B. trematum* isolates detected between August 2019 and January 2020 to aid the matter. Cryo-conserved isolates were subcultured and re-identified using various routine means of identification. Bacterial genomes were fully Illumina-sequenced and phenotypical susceptibility was determined by broth microdilution and gradient-strip tests. All isolates displayed increased susceptibility to piperacillin–tazobactam (<2/4 mg/L), imipenem (<1 mg/L), and meropenem (<0.047 mg/L), whereas they displayed decreased susceptibility to all tested cephalosporins and fluoroquinolones (according to PK-PD, EUCAST 10.0 2020). One isolate carried a beta-lactamase (EC 3.5.2.6) and a sulfonamide resistance gene (sul2) and cells displayed resistance to ampicillin, ampicillin/sulbactam, and trimethoprim/sulfamethoxazole. All isolates carried genes conferring decreased susceptibility to aminoglycosides (aadA), fosfomycin (fosA) and fluoroquinolones (gyrB EC 5.99.1.3). Awareness that *B. trematum* can be resistant to trimethoprim/sulfamethoxazole is warranted.

## 1. Introduction

*Bordetella trematum* is a relatively newly discovered [1] Gram-negative, encapsulated, nonsporulating rod in the family of the *Alcaligenaceae* that belongs to the taxonomically heterogeneous group of the non-fermenters and as such does not rely on sugars for energy production. While its fastidious growth and barely visible colonies hamper its detection, once detected, biochemical identification systems frequently misidentify it, most commonly as *Bordetella avium*, *Bordetella bronchiseptica*, or *Achromobacter denitrificans* [2,3,4]. To date a reliable accurate discrimination in laboratory routine is only possible with matrix-assisted laser desorption ionization time-of-flight (MALDI-TOF) mass spectrometry that should further be confirmed by 16s rRNA gene sequencing.

Nonfermenting Gram-negative bacilli have emerged as important healthcare-associated pathogens [5]. The most important ones in this regard are *Pseudomonas aeruginosa*, *Acinetobacter baumannii,* and *Stenotrophomonas maltophilia*. A recent literature review reveals the clinical significance of *B. trematum* to still be a matter of debate as the little information available on this pathogen left authors largely uncertain as to whether therapy was necessary or superfluous in the respective cases [2,3,4,6,7,8,9,10,11,12,13]. Previously documented cases have mainly been isolated from ulcers and soft tissue infections, even though occasional cases of bacteremia have been described [6,7]. Given the limited amount of documented cases, each new documented case adds evidence to the issue. We contribute to the matter by providing an in-depth analysis of three unique clinical *B. trematum* isolates, detected in wound swabs of three patients of the University Hospital Bonn between December 2019 and April 2020 and hope to aid the matter by being among the first to provide molecular susceptibility profiles of clinical *B. trematum* isolates that explain and supplement previously documented and current phenotypical susceptibility-patterns.

## 2. Results

### 2.1. Origin of the Isolates

The three *Bordetella trematum* isolates were isolated from wound swabs of patients of the University Hospital Bonn between December 2019 and April 2020. Isolate one belonged to a 75-year-old female patient with chronic renal insufficiency, arterial hypertension, and hypothyroidism, and was detected in a wound swab of an ulcer on the right heel, together with *Staphylococcus aureus* and *Enterobacter cloacae* complex. Isolate two belonged to a 49-year-old obese male patient with hereditary factor V Leiden, post-thrombotic syndrome, chronic ulcerations, chronic obstructive pulmonary disease, and nicotine abuse, and was the only bacterial agent to be detected in a wound swab of an ulcer on the left foot. Isolate three belonged to an 81-year-old male patient with peripheral arterial disease and peripheral neuropathy and was detected in a tissue sample of the left distal tibia together with *Staphylococcus capitis*.

### 2.2. Identification

Identification by MALDI-TOF MS was successful with both systems (Vitek MS and Bruker). The Vitek 2 GN ID Card identified isolate one once as *Acinetobacter lwoffii* (95%) and twice as *Bordetella hinzii*, isolate two twice as *B. hinzii* and once completely failed to identify it, and isolate three once as *A. lwoffii* (50%) or *B. trematum* (50%), once as *B. hinzii* (34%), *A. lwoffii* (33%), or *Alcaligenes faecalis* (33%), and once as *A. faecalis* (51%), or *B. hinzii* (49%). Ribosomal 16s DNA sequencing and WGS confirmed all three isolates as being *B. trematum*. All three isolates tested oxidase positive.

### 2.3. Susceptibility Testing

The results of the susceptibility testing by gradient strip and automated dilution are displayed in Table 1. *B. trematum* isolates one and three were very similar in terms of their antibiotic susceptibility profile. All three isolates were highly susceptible to piperacillin–tazobactam and all tested carbapenems. All three isolates tested resistant or decreasingly susceptible to most tested cephalosporins and fluoroquinolones. Isolate two displayed decreased susceptibility or resistance to ampicillin, ampicillin/sulbactam, and trimethoprim/sulfamethoxazole. Small colony variation was determined to be unlikely based on colony morphology.

### 2.4. Genome Analysis

The ResFinder of the CGE Server [14,15] detected one acquired antimicrobial resistance gene in isolate two, namely sul2, with 100% identity, conferring resistance to sulphonamides. No other acquired resistance genes present in the database were detected. The gene annotation server RAST [16,17,18] identified genes conferring decreased susceptibility to aminoglycosides (aadA), fosfomycin (fosA), and fluoroquinolones (gyrB EC 5.99.1.3) on the genomes of all three isolates. On the genome of isolate two additionally a gene encoding for a beta-lactamase (EC 3.5.2.6) was detected. The latter was not encoded on the same contig as the sul2 gene. The number of annotated genes per subsystem for each isolate is reported in Table 2.

## 3. Discussion

In 1996 *Bordetella trematum* was described as being the only oxidase-negative *Bordetella* species [1]. Later, however, the emergence of oxidase-positive isolates [8] was reported, questioning reliability of this tool for biochemical differentiation. Reasons for this incongruity were hypothesized to lie in the different derivatives used for the oxidase reaction in the two studies [2], *N,N″*-dimethyl-p-phenylene monochloride in the earlier and *N,N,N′,N′*-tetramethyl-p-phenylenediamine dihydrochloride in the latter study. Our isolates tested positive with the latter reagent.

Colony morphology of *B. trematum* can result in misidentification as coagulase-negative staphylococci. Its most frequent location in clinical practice being chronic wounds, resembling skin microbiota, a rather marginally significant finding in this context, which makes it prone to overlooking. Given that *B. trematum* is then also frequently misidentified when identification is performed by means other than MALDI-TOF MS or sequencing, a significant number of isolates might currently be missed. Biochemical identification systems frequently misidentify it as *Bordetella avium*, *Bordetella bronchiseptica*, or *Achromobacter denitrificans* [2,3,4]. In our study, biochemical identification systems misidentified *B. trematum* as *Bordetella hinzii*, *Acinetobacter lwoffii*, and *Alcaligenes faecalis*. Hence, we postulate this organism to be more prevalent than currently assumed and it to be more frequently detected in the future, if biochemical identification means will be largely replaced by mass spectrometry and DNA sequencing.

*B. trematum* is generally isolated from necrotic lesions or sites with impaired tissue perfusion such as chronic ulcers, even though multiple reports have emerged highlighting the organism’s capability of invading the bloodstream, leading to bacteremia and even sepsis [6,7,8,9]. None of our three clinical *B. trematum* isolates caused invasive disease. While surgical debridement surely represents the treatment of choice for most chronic ulcers, systemic *B. trematum* infection requires antimicrobial therapy. Generally, poly-microbial infections of chronic wounds are always best tackled by an interdisciplinary approach, involving surgeons as well as microbiologists.

Chronic, non-healing wounds are frequently hypoxic [19]. Cell death, tissue necrosis, and an impaired immune response, all linked to decreased tissue perfusion, create ideal growth conditions for a large number of microorganisms, thus explaining the poly-microbial nature of such infections [20]. Since microorganisms from a variety of sources are presented with an opportunity to colonize this unnatural habitat, microbial interactions unique to this particular environment may significantly influence wound pathogenesis and healing [21]. Antimicrobial treatment that covers potentially synergistic microorganisms such as *B. trematum* in addition to known pathogens should be considered in cases in which improvement stays out after appropriate antimicrobial coverage of the latter. *B. trematum* is frequently isolated along with other agents, most commonly *S. aureus*, *Enterobacterales* and other non-fermenters [2,3,8,10,11]. Nevertheless, as was the case with one of our isolates, cases in which *B. trematum* is the only agent isolated from a clinical specimen do occur [7,13]. Given the higher likelihood for chronic wounds in patients with diabetes and peripheral arterial and/or cardiovascular disease, these patients have been prominently featured, among those detected with *B. trematum*. All three of our isolates belonged to patients with underlying cardiovascular disease; however, none had a diagnosis of diabetes.

As regards documented *B. trematum* antibiotic susceptibility profiles we see some similarities. Except in one case [13], in which identification occurred by unstandardized means and the susceptibility profile rendered the results even more questionable, all *B. trematum* isolates described in the literature were susceptible to tested carbapenems and piperacillin-tazobactam and showed markedly decreased susceptibility to 2nd and 3rd generation cephalosporins and fluoroquinolones. High MIC-values for aminoglycosides and aminopenicillins [2,6,10,13] were not uncommon. That we identified genes conferring decreased susceptibility to aminoglycosides, fosfomycin, and fluoroquinolones on the genomes of all three isolates, together with the observation that most if not all isolates described in the literature showed decreased susceptibility to these substances, suggests that such genes are common among *B. trematum* strains. To our knowledge, we describe the first documented *B. trematum* isolate phenotypically resistant to trimethoprim/sulfamethoxazole and detected the sul-2 gene on its genome, likely responsible for this resistance encoding forms of dihydropteroate synthase that are not inhibited by the substance, and thus invite to rethink the assumption that *B. trematum* can safely be considered as susceptible to this substance [12].

We also detected the same isolate to carry a beta-lactamase-encoding gene. The capability of *B. trematum* to carry and express such genes could explain why some described isolates display high MIC-values for aminopenicillins.

This study has several limitations. It is a single center study with three isolates. The two susceptibility-testing systems were commercially available test systems and not the recommended in-house systems; accuracy of these certified test systems should, however, be comparable to in-house systems. No specific breakpoints exist for the interpretation of the minimal inhibitory concentration, hence interpretation followed EUCAST PK/PD breakpoints.

## 4. Materials and Methods

After completion of the routine diagnostic procedures the isolates were cryo-conserved at −78 °C. For this study the isolates were thawed and sub-cultured twice on Columbia 5% sheep blood agar (Becton Dickinson, Heidelberg, Germany) prior to testing.

### 4.1. Identification

Isolates were identified thrice by matrix-assisted laser desorption ionization time-of-flight mass spectrometry (MALDI-TOF MS) by Vitek MS (bioMerieux, Marcy-l’Etoile, France, software version 4.7.1) and Bruker (Bruker Daltonik, Bremen, Germany, software version 3.1). Additionally, the Vitek 2 GN ID Card (bioMerieux, Marcy-l’Etoile, France, Vitek 2 software version 4.7.1) was used thrice for identification. Identification was further verified by 16s ribosomal DNA sequencing (Sanger, Microsynth, Balgach, Switzerland; NCBI). For 16s DNA sequencing, DNA was isolated with the Wizard Genomic DNA Purification Kit (Promega, Madison, WI, USA). The oxidase was determined with BD BBL Dry Oxidase Slide (*N,N,N′,N′*-tetramethyl-p-phenylenediamine dihydrochloride) (Becton Dickinson, Sparks, NV, USA).

### 4.2. Susceptibility Testing

Susceptibility of all isolates was determined thrice by broth microdilution and gradient strip. Susceptibility tests were employed strictly according to the manufacturer’s instruction. From each isolate, a bacterial suspension in 0.9% saline solution was prepared. The suspension was adjusted to a McFarland value of between 0.48 and 0.52 using a DensiCHEK plus photometer (bioMerieux, Marcy-l’Etoile, France). For the gradient strip test, MIC Test Strips (Liofilchem, Roseto degli Abruzzi, Italy) were used (except for piperacillin-tazobactam (bioMerieux, Marcy-l’Etoile, France)) and were carried out on Mueller–Hinton agar plates (Becton Dickinson, Heidelberg, Germany). MIC was visually read from the gradient strip and was rounded up to the next standard upper two-fold serial dilution value. For the broth microdilution, MIC-Plates Micronaut-S MDR MRGN-Screening and Micronaut Sepsis GN (Merlin, Bornheim, Germany) were taken. Tests were performed with Mueller–Hinton broth (Merlin, Bornheim, Germany) and were visually read. All measurements were continuously supervised head-on by two medical doctors. Each sample was visually and independently read by three trained observers. MICs were interpreted according to EUCAST PK-PD breakpoints (version 10.0, 2020). Small colony variation was evaluated by daily subcultivating cultures over a two-week-long period.

### 4.3. Sequencing and Analysis

Highly purified DNA was extracted from all strains using the column-based DNeasy UltraClean Microbial Kit (Qiagen GmbH, Hilden, Germany). The isolation was performed according to the manufacturer’s instructions with the exception that at the end of the extraction process the DNA was eluted to 100 μL volume. The obtained DNA was qualitatively and quantitatively evaluated using the NanoDrop OneC from Thermo Fisher Scientific Inc. (Waltham, MA, USA) Dual-indexed Illumina sequencing libraries were constructed from each sample using the NexteraXT kit (Illumina, San Diego, CA, USA), pooled, and sequenced on the Illumina MiSeq platform (Illumina, San Diego, CA, USA). Raw reads have been uploaded to the Sequence Read Archive (SRA); accession PRJNA747254. For de novo assembly, paired-end reads were trimmed and filtered with BBDuk Trimmer, a Q value of 20 was chosen. De novo assembly was then performed using Geneious Prime (software version 2020.1 Biomatters, Auckland, New Zealand) and multi-contig draft genomes (<150 contigs per isolate; N50 varied between 78,865 and 113,381) were generated for each isolate, by Mauve-aligning the contigs to the reference genome sequence NZ CP016340.1 of *Bordetella trematum* strain F581 and subsequent concatenation. Genome analysis was performed with software tools of the CGE Server (Update 8 June 2020, Center for Genomic Epidemiology, DTU, Denmark). Complete bacterial genome annotation was performed using RAST (Annotation scheme RASTtk).

## 5. Conclusions

Thanks to technological progress in the identification of microorganisms *Bordetella trematum*, mainly isolated from chronic open ulcers and wounds on the lower extremities, is being increasingly identified. As its role in the pathogenesis of these conditions does not appear to always be causative by itself, antibiotic treatment might not be necessary in every patient, but can improve the outcome in some. In synopsis with previously documented cases our study shows that identifications of *B. trematum* are increasing. In cases in which antibiotic treatment is necessary, awareness that *B. trematum* can be resistant to trimethoprim/sulfamethoxazole is warranted.

## Figures and Tables

**Table 1:** Antibiotic susceptibility profile of the three *B. trematum* strains under study. Results (in mg/L) of the broth microdilution displayed on the left and of the gradient-strip diffusion on the right of the table. Concentrations highlighted in green indicate the isolate would be considered as susceptible to it, in yellow susceptible at high dosage, and in red resistant according to PK-PD, EUCAST 10.0 2020.

	Isolate 1	Isolate 2	Isolate 3	Isolate 1	Isolate 2	Isolate 3
Ampicillin	<2	>16	<2	2	>256	3
Ampicillin/Sulbactam	<2/4	>16/4	<2/4	1.5	6	1.5
Amikacin	<4 *	<4 *	<4 *			
Cefepime	2	8	4			
Ceftriaxone				3	4	4
Cefotaxime	>2	>2	>2			
Ceftazidime	4	4	4	4	6	4
Ceftazidime/Avibactam	4/4	4/4	4/4			
Ceftolozane/Tazobactam	>8/4	>8/4	>8/4			
Chloramphenicol	16 **	16 **	16 **			
Ciprofloxacin	2	2	2	1.5	1.5	1
Colistin	<1 **	<1 **	<1 **			
Fosfomycin	>64 **	>64 **	>64 **			
Gentamicin	<1	2	2			
Imipenem	<1	<1	<1			
Levofloxacin	1	1	1			
Meropenem	<0.125	<0.125	<0.125	0.047	0.047	0.047
Piperacillin	<8 *	<8 *	<8 *			
Piperacillin/ Tazobactam	<2/4	<2/4	<2/4	0.75	1.5	0.75
Temocillin	128 **	128 **	128 **			
Tigecycline	<0.25	<0.25	<0.25			
Tobramycin	<1 *	<1 *	2			
Trimethoprim/Sulfamethoxazole	<1/19 **	>4/76 **	<1/19 **	0.094 **	>32 **	0.125 **

* Applied method not sufficiently accurate for the evaluation of the susceptibility profile according to PK-PD for this substance; ** Insufficient evidence that the organism is a good target for therapy with the agent according yo EU-CAST 10.0.2020.

**Table 2:** Number of annotated genes per subsystem of the three *B. trematum* strains detected by the gene annotation server RAST in regard to antibiotic resistance and invasion.

Subsystem Feature Counts	Isolate	B1	B2	B3
**Antibiotic/** **Toxin Res.:**	Copper homeostasis	8	7	7
	Bile hydrolysis	2	2	2
	Cobalt-zinc- cadmium res.	5	6	5
	Aminoglycoside adenylyltr.	1	1	1
	Copper tolreance	3	3	3
	Fosfomycin resistance	1	1	1
	Fluoroquinolone resistance	2	2	2
	Beta-lactamase	0	1	0
	Chromioum compound res.	1	2	1
**Invasion/** **Intracellular Res.:**	SSU ribosomal proteins	4	4	4
	Mycob. Virulence operon inv. in DNA transcr.	2	2	2
	LSU ribosomal proteins	3	3	3

## Data Availability

Raw reads have been uploaded to the Sequence Read Archive (SRA); accession PRJNA747254.
